# Heterozygosity of ALG9 in Association with Autosomal Dominant Polycystic Liver Disease

**DOI:** 10.3390/genes14091755

**Published:** 2023-09-02

**Authors:** Melissa M. Boerrigter, Renée Duijzer, René H. M. te Morsche, Joost P. H. Drenth

**Affiliations:** 1 Department of Gastroenterology and Hepatology, Research Institute for Medical Innovation, Radboud University Medical Center, 6525 GA Nijmegen, The Netherlands; 2 European Reference Network RARE-LIVER, D-20246 Hamburg, Germany

**Keywords:** ADPLD, ALG9, whole exome sequencing, polycystic liver disease (PLD), PCLD, cyst wall

## Abstract

α-1,2-mannosyltransferase (*ALG9*) germline variants are linked to autosomal dominant polycystic kidney disease (ADPKD). Many individuals affected with ADPKD possess polycystic livers as a common extrarenal manifestation. We performed whole exome sequencing in a female with autosomal dominant polycystic liver disease (ADPLD) without kidney cysts and established the presence of a heterozygous missense variant (c.677G>C p.(Gly226Ala)) in *ALG9*. In silico pathogenicity prediction and 3D protein modeling determined this variant as pathogenic. Loss of heterozygosity is regularly seen in liver cyst walls. Immunohistochemistry indicated the absence of ALG9 in liver tissue from this patient. ALG9 expression was absent in cyst wall lining from *ALG9*- and *PRKCSH*-caused ADPLD patients but present in the liver cyst lining derived from an ADPKD patient with a *PKD2* variant. Thus, heterozygous pathogenic variants in *ALG9* are also associated with ADPLD. Somatic loss of heterozygosity of the ALG9 enzyme was seen in the *ALG9* patient but also in ADPLD patients with a different genetic background. This expanded the phenotypic spectrum of ADPLD to *ALG9*.

## 1. Introduction

The folding of polypeptides in the endoplasmic reticulum (ER) contributes to properly structured and functional proteins. N-linked glycosylation is the co-translational modification step where an oligosaccharide chain is transferred to the asparagine (N) residues of the polypeptide [1]. Glycosylation and trimming of the oligosaccharide chain are essential to promote and maintain proper protein folding and quality control [1,2]. One of the enzymes essential for the formation of the oligosaccharide chain is the α-1,2-mannosyltransferase ALG9 encoded by *ALG9*. In the ER, ALG9 adds a mannose group at two positions of the oligosaccharide chain [3]. Deficiency of ALG9 results in hypoglycosylation and increased degradation of misfolded glycoproteins [4,5].

Autosomal recessive loss of function variants in *ALG9* cause congenital disorder of glycosylation type IL (CDG-IL or ALG9-CDG (OMIM #608776), or Gillessen–Kaesbach–Nishimura syndrome (OMIM #263210)). To date, nineteen patients with homozygous pathogenic variants in *ALG9* have been reported [4,5,6,7,8,9,10,11]. These patients presented with a wide range of clinical phenotypes [4,5,6,7,8,9,10,11]. Five different variants have been associated with this CDG, and patients presented with facial dysmorphism, muscular hypotonia, epileptic seizures, developmental delay, cardiac failure, and skeletal dysplasia [10]. Renal cysts and mild to moderate hepatomegaly were observed in 5 out of 15 and 9 out of 13 patients, respectively [10].

Heterozygous pathogenic variants in *ALG9* have been detected in patients with autosomal dominant polycystic kidney disease (ADPKD) [12,13]. In patients with ADPKD, there is a gradual formation and increase in the size of kidney cysts often associated with renal function loss. ADPKD may be accompanied by polycystic liver disease (PLD), a condition arbitrarily defined as >10 liver cysts [14,15]. In the absence of renal cysts, PLD is labeled as autosomal dominant polycystic liver disease (ADPLD). In contrast to polycystic kidneys that negatively affect renal functioning, a polycystic liver does not affect the function of the liver [15,16]. Most PLD patients are asymptomatic [17]. However, due to the increase in cyst volume or the number of cysts, hepatomegaly can result in abdominal pain, early satiety, gastroesophageal reflux, indigestion, nausea, shortness of breath, and abdominal wall hernias [15,18]. Due to these symptoms, an ADPLD individual’s quality of life (QoL) may be compromised [19,20]. Liver transplantation is the only definitive cure for PLD, but only ~1.4% of liver transplants are directed to PLD individuals [21,22].

Other available treatment options, such as surgical fenestration or treatment with somatostatin analogues, are aimed at an individual cyst or total liver volume reduction, respectively [15,16,23,24].

ADPKD and ADPLD are genetic diseases caused by a heterozygous germline variant in a wide range of genes. ADPKD is primarily caused by a pathogenic variant in polycystin-1 (*PKD1*) or polycystin-2 (*PKD2*) [16,24,25]. However, there is a small number of individuals in whom a pathogenic variant in dolichyl-phosphate glucosyltransferase (*ALG5*), DnaJ homolog subfamily B member 11 (*DNAJB11*), or intraflagellar transport protein 140 homolog (*IFT140*) has been established as the cause of ADPKD [26,27,28,29,30].

In the majority of ADPLD cases, ADPLD is attributed to pathogenic variants in either glucosidase II subunit β (*PRKCSH*) or translocation protein SEC63 homolog (*SEC63*) [16,24,25,31]. In these cases, individuals primarily develop PLD but do not or only rarely develop kidney cysts [16,24,25,31]. In addition, there are a number of genes that, when affected, cause ADPLD in a small number of patients. These genes are α-1,3-glucosyltransferase (*ALG8*), Bardet–Biedl syndrome 4 (*BBS4*), fibrocystin (*PKHD1*), and protein transport protein SEC61 subunit β (*SEC61B*) [32,33,34,35,36]. Abnormal function of the encoded proteins may cause polycystic livers with a very wide range of severity [32,33,34,35,36]. Most of these latter cases with heterozygous pathogenic variants in these minor genes may also present with a small number of kidney cysts that do not alter renal function and the number of cysts also does not meet formal ADPKD criteria [32,33,34,35,36].

The discovery of pathogenic variants in genes responsible for the development of both diseases helped to better understand the potential molecular mechanism of polycystic liver disease, but on the other hand, the genetic complexity of polycystic liver disease is evident. Some genes are found to cause polycystic kidneys without liver cysts (ADPKD), polycystic kidneys with a polycystic liver (ADPKD with PLD), a polycystic liver with a small number of kidney cysts (ADPLD with kidney cysts), and a polycystic liver without kidney cysts (ADPLD) [31,32,37,38,39,40,41,42,43]. For example, the clinical spectrum in individuals with a pathogenic variant in glucosidase II subunit α (*GANAB*), low-density lipoprotein receptor-related protein 5 (*LRP5*), and protein transport protein Sec61 subunit α isoform 1 (*SEC61A1*) is very large [31,32,37,38,39,40,41,42,43]. It is to be expected that the number of genes associated with ADPLD or ADPKD will increase.

Most polycystic liver- and polycystic kidney-related proteins are located in the primary cilium and the ER. Polycystin-1 and polycystin-2, encoded by *PKD1* and *PKD2*, localize to the primary cilium and are involved in calcium signaling, mechanotransduction, and cell proliferation [15,16,24]. Fibrocystin (*PKHD1*) is speculated to be involved in developing and maintaining the primary cilium morphology [15,16,24]. IFT140 and BBS4 are part of complexes that play central roles in the movement of particles through the primary cilium [30,35]. LRP5 is involved in the canonical WNT–β-catenin pathway, a signaling pathway debated to affect ciliogenesis [15,16,24].

The other polycystic liver and polycystic kidney disease proteins localize to the ER. The proteins encoded by *SEC61A*, *SEC61B*, and *SEC63* transport newly translated polypeptides into the ER [15,16,24,43]. DNAJB11 assists in the stabilization of the polypeptides during this transport but also controls protein folding during the addition of the oligosaccharide chains that are produced by the *ALG5*, *ALG8*, *ALG9*, and many other enzymes essential for N-linked glycosylation [15,16,24]. The proteins encoded by *PRKCSH* and *GANAB* form a protein complex that is involved in the trimming of the polypeptide-bound oligosaccharide chains and trigger the quality control of the folded polypeptide before SEC61 transports incorrectly translated proteins out of the ER [15,16,24]. Pathogenic variants in any of these genes can lead to aberrant protein processing and potentially trigger cystogenesis in the liver. Differentiation between ADPKD and ADPLD can be complicated in view of the overlapping phenotypes. However, the distinction between these phenotypes is important for disease management [13,25]. This is because patients with polycystic kidneys or individuals who are more likely to develop progressive kidney cysts require more medical monitoring to track the development of potential kidney failure [25,44].

A pathogenic variant in *PRKCSH* or *PKD2* always causes ADPLD or ADPKD, respectively, without a phenotypic overlap between the two diseases [24,25]. This case report describes a patient with ADPLD without kidney involvement who carries a novel heterozygous pathogenic variant in *ALG9*. The case description aims to illustrate the expanding phenotype of *ALG9* and the complexity of genotype–phenotype differentiation in ADPKD and ADPLD.

## 2. Materials and Methods

### 2.1. Patient Selection

Clinically diagnosed ADPLD patients without a previous genetic diagnosis were approached for genetic screening by whole exome sequencing (WES). ADPLD was defined as the presence of more than ten liver cysts upon medical imaging (magnetic resonance imaging (MRI) or computed tomography (CT)) [15]. Patients provided whole blood and written informed consent for WES.

In order to compare potential variations in ALG9 expression due to the differences in molecular mechanisms between ADPLD and ADPKD, we compared this case with two PLD patients with genetic diagnoses in the major PLD genes *PRKCSH* for ADPLD and *PKD2* for ADPKD. The control ADPLD patient was diagnosed with a frequently appearing heterozygous pathogenic splice site variant in *PRKCSH* (c.292+1G>C p.? (NM_001289104.2)) during diagnostic screening. The control ADPKD with PLD patient was diagnosed with a heterozygous pathogenic frameshift variant in *PKD2* (c.2584del p.(Ala862Profs*2) (NM_000297.4)) during research screening [45]. Both control patients provided informed consent for PLD-related research.

### 2.2. Genetic Screening

Experiments started with the isolation of genomic DNA from whole blood samples, following the established protocol of the High Pure PCR Template Preparation Kit (11796828001, Roche Life Science, Penzberg, Germany). Subsequently, DNA underwent enrichment using the Twist Human Core Exome Kit (104136, Twist Bioscience, South San Francisco, CA, USA). WES was performed through 2 × 150 base pairs end sequencing using a NovaSeq 6000 Sequencing System (Illumina, San Diego, CA, USA). The obtained sequence reads were aligned to the GRCh37/hg19 human reference genome using the Burrows–Wheeler Aligner [46]. Variants meeting the following criteria were chosen: variant variation between 25–75%, non-synonymous, minor allele frequency ≤0.001 in a population database (GnomAD [47], ESP [48], or GoNL [49]), CADD score ≥20, deleterious by at least one prediction program (SIFT [50], MutationTaster [51], or PolyPhen-2 [52]), and exonic variant or splice site variant ≤10 base pairs from the splice site. Variants were confirmed according to the standard Sanger sequencing protocol with the Big Dye Terminator v1.1 Cycle Sequencing Kit (4337452, Thermo Fisher Scientific, Waltham, MA, USA) and the 3730XL DNA Analyzer (Applied Biosystems, Waltham, MA, USA).

Pathogenicity of variants was determined using the American College of Medical Genetics and Genomics/American Association of Molecular Pathology (ACMG/AMP) classification guidelines [53] and Alamut Visual Plus (version 1.4, SOPHiA GENETICS, Bidart, France) in December 2022.

### 2.3. Conservation Analysis

Homology sequence alignment was performed with the multiple sequence alignment tool PRALINE (Centre of Integrative Bioinformatics, Vrije Universiteit Amsterdam, The Netherlands) [54].

### 2.4. 3D Modeling

Structural effects were analyzed with project HOPE (CMBI, Radboud University Nijmegen, The Netherlands) [55]. The 3D structures were constructed based on the prediction of the human ALG9 (Q9H6U8) by AlphaFold DB (version 1 July 2021) (EMBL-EBI, Hinxton, UK) and the visualization program YASARA (YASARA Biosciences/Bio-Prodict/WHAT IF Foundation, Vienna, Austria and Nijmegen, The Netherlands) [56,57,58].

### 2.5. Fluorescent Immunohistochemistry

Formalin-fixed, paraffin-embedded tissue sections (4 µm) were blocked with a blocking buffer containing 1% normal swine serum blocking solution (Vector Laboratories, Burlingame, CA, USA), 1% bovine serum albumin (BSA) (Sigma-Aldrich, Burlington, MA, USA), and 0.1% gelatin from cold-water fish skin (Sigma-Aldrich) in 1× Gibco phosphate-buffered saline (PBS) (Thermo Fisher Scientific). Sections were incubated overnight with the primary antibodies anti-CK19 (mouse, 1:200, MU246-UC, BioGenex, Fremont, CA, USA) and anti-ALG9 (rabbit, 1:200, HPA038575, Sigma-Aldrich) at 4 °C. Secondary antibodies FITC 490 anti-rabbit (goat, 1:200, 111-005-003, Jackson ImmunoResearch, West Grove, PA, USA) and TexasRed 570 anti-mouse (goat, 1:200, 115-075-062, Jackson ImmunoResearch) were incubated for 45 min at room temperature. Protein expression and localization were visualized with the Zeiss Imager Z2 microscope combined with the ApoTome.2 (Zeiss, Oberkochen, Germany) and analyzed with ZEN 2 blue (version 10, Zeiss).

### 2.6. Ethics Approval

This protocol was met with approval by the Dutch Institutional Review Board and Ethics Committee (CMO Arnhem-Nijmegen; 2020-6326). All patients granted informed consent.

## 3. Results

### 3.1. Clinical Characteristics

A 60-year-old female with a medical history of umbilical hernia and diaphragmatic hernia visited our outpatient clinic with gastroesophageal reflux complaints and abdominal discomfort. The patient gave a negative family history for liver or kidney cysts.

**Figure 1:**
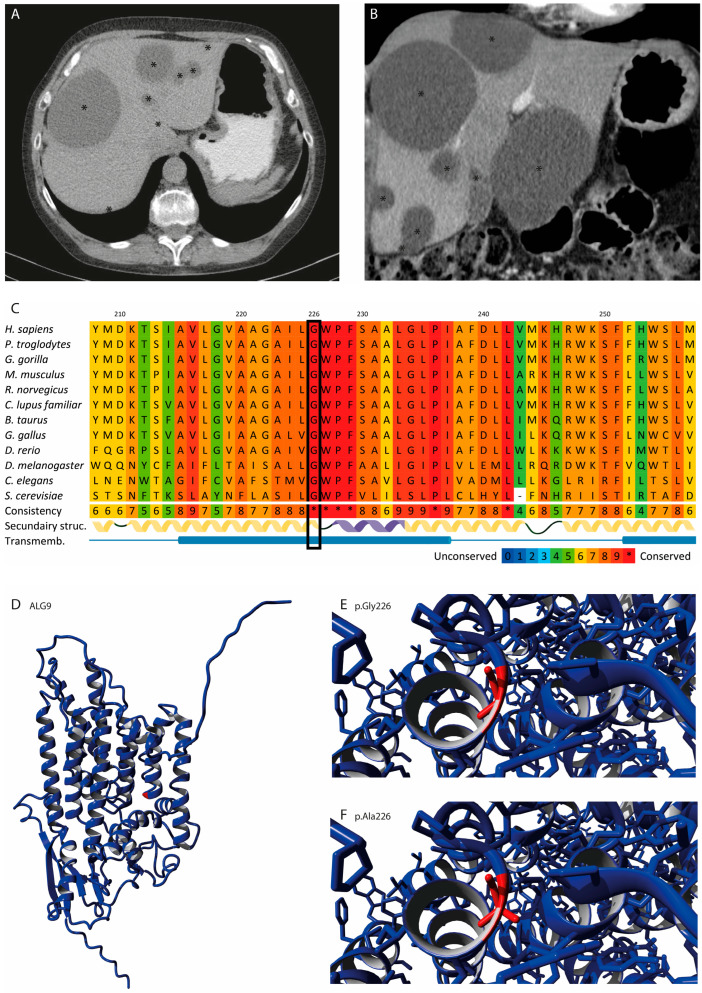
**Phenotype and variant interpretation of *ALG9* c.677G>C.** (**A**,**B**) Transverse and coronal CT scans of the patient before surgery. On CT scans, liver cysts are displayed as homogenous, darker-gray circular shapes. The locations of the liver cysts are marked with black asterisks (*). The displayed cross-sectional images were chosen to illustrate cyst distribution and volume, not to illustrate the number of cysts. (**C**) Conservation analysis in 12 species with ALG9′s secondary structure (yellow: α helix; purple: 3_10_ helix; green: coil) and transmembrane regions (blue). (**D**) The 3D structure of wildtype ALG9 with amino acid position 226 in red. (**E**) Close-up of glycine at amino acid position 226 in red. (**F**) Close-up of alanine at amino acid position 226 in red.

The CT scan showed diffuse multiple liver cysts (>10), but kidney cysts were absent, compatible with a diagnosis of ADPLD (Figure 1A,B). The diameters of most liver cysts ranged from 1 to 5 cm. Additionally, two dominant cysts with diameters of 7 cm and 11 cm were present in segment 7 and segments 2 and 3, respectively. This led to a total liver volume of 2.7 L. The largest cyst in segments 2 and 3 was treated with aspiration sclerotherapy, but due to insufficient symptom relief, successful laparoscopic fenestration followed within 6 months. After a follow-up of five years, an ultrasound showed no recurrence of dominant cysts, and the patient reported no development of cyst-related complaints.

The first control patient was a 58-year-old female with a medical history of asthma and ADPKD, with a mild renal phenotype but severe hepatomegaly (9.2 L) due to innumerable cysts in the liver. In the past, she was treated with lanreotide in a study context. This treatment had a minimal effect on liver volume and complaints. The ADPKD was diagnosed to be caused by a heterozygous pathogenic frameshift variant in *PKD2*.

The second control patient was a 50-year-old female with severe ADPLD. She had a history of multiple liver cyst ruptures, and despite treatment with lanreotide in a study context and two laparoscopic fenestration procedures, her total liver volume remained large (8.5 L). The innumerable cysts in the liver and the lack of cysts in the kidney were found to be due to a heterozygous pathogenic splice site variant in *PRKCSH*.

### 3.2. Pathogenicity Prediction

Previous targeted mutational analysis of the ADPLD genes *PRKCSH* and *SEC63* yielded no pathogenic variants or variants of unknown significance. WES revealed a heterozygous germline variant in the cyst-associated gene *ALG9* in our patient. The missense variant c.677G>C p.(Gly226Ala) (NM_024740.2, Chr11(GRCh38):g.111857626C>G) was likely pathogenic, based on the ACMG/AMP classification guidelines, and not previously registered in the population databases GnomAD, ESP, or GoNL. The glycine at amino acid position 226 of ALG9 was highly conserved through the species (Figure 1C) and positioned in the mannosyltransferase domain (Figure 1D). The side chain of the glycine was usually positioned on the inside of the α helix, which is part of one of the transmembrane domains (Figure 1E). The side chain of alanine was slightly bigger, more hydrophobic, and less flexible (Figure 1F). This alteration will affect the conformation of the local backbone and disturb the local structure. The difference in amino acid size and hydrophobicity may affect membrane lipids and disturb protein function.

### 3.3. ALG9 Expression in the Cyst Wall Lining

**Figure 2:**
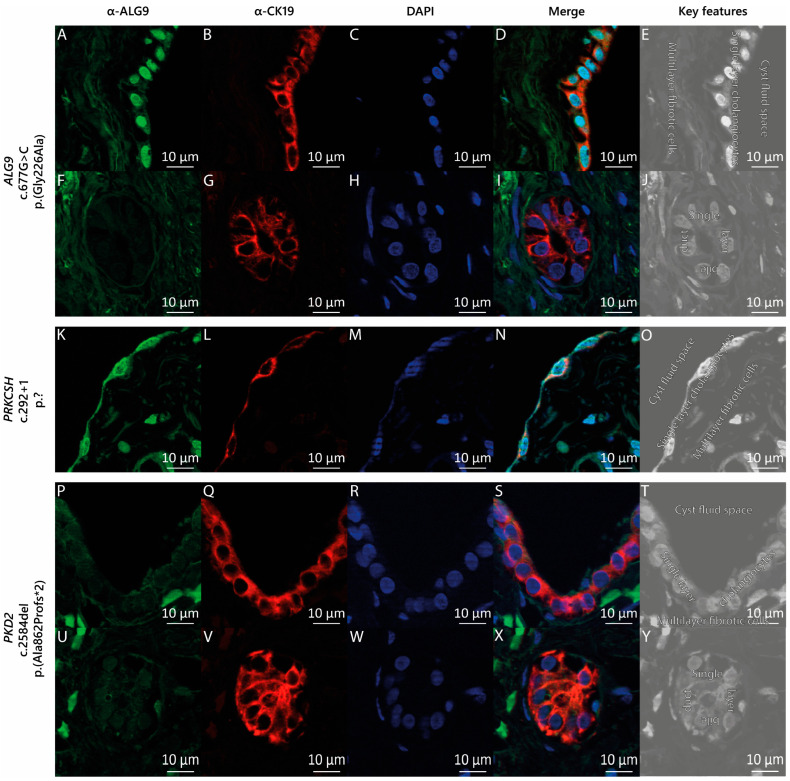
**ALG9 expression in cystic liver tissue.** (**A**–**J**) ADPLD individual with the heterozygous pathogenic missense variant in *ALG9*, (**K**–**O**) an ADPLD individual with a heterozygous pathogenic splice site variant in *PRKCSH*, and (**P**–**Y**) an ADPKD individual with PLD with a heterozygous pathogenic frameshift variant in *PKD2*. Green: protein of interest marker ALG9; red: cholangiocyte marker CK19; blue: DNA marker DAPI. The key features of liver cyst tissue: The epithelium lining of a liver cyst is a single-cell layer of cholangiocytes, which encapsulates the cyst fluid and is generally surrounded by a multilayer of fibrotic/connective cells (**E**,**O**,**T**). The epithelium lining of a bile duct is also a single-cell layer of cholangiocytes. However, the epithelial cells of the bile duct are more cuboidal shaped (**J**,**Y**). Image magnification: 63×.

The liver cyst specimen from this patient was derived through laparoscopic fenestration. Histology of the lining epithelium of the cyst displayed columnar epithelium compatible with cholangiocytes. Immunostaining with CK19 demonstrated the diffuse positive expression of CK19 in the cyst wall lining (Figure 2B) and the bile duct lining (Figure 2G). We then stained liver cyst tissue from our patient with the *ALG9* c.677G>C p.(Gly226Ala) variant with anti-ALG9 antibodies (Figure 2A,F). We compared the ALG9 staining pattern (Figure 2A–J) with that of the ADPLD patient carrying a heterozygous germline variant in *PRKCSH* (c.292+1 p.?) (Figure 2K–O) and that of the ADPKD patient carrying a heterozygous germline variant in *PKD2* (c.2584del p.(Ala862Profs*2)) (Figure 2P–Y). We found that ALG9 was present in the cyst wall lining of both ADPLD patients (Figure 2A,K) but absent from the cystic wall lining of the ADPKD patient with the *PKD2* (c.2584del) variant (Figure 2P). We also discovered that in our *ALG9* and *PKD2* carriers, ALG9 was not expressed in the bile ducts (Figure 2F,U). Figure 2E,J,O,T,Y displays the characteristic key features of cystic liver tissue.

## 4. Discussion

In this study, we identified an ADPLD patient without kidney cysts with a heterozygous pathogenic variant in *ALG9*. In silico pathogenetic analyses predicted that this missense variant was pathogenic. Protein expression analysis with the available patient materials showed that ALG9 was expressed in the cystic wall lining of ADPLD patients but not ADPKD patients.

*PKD1* and *PKD2* variants are linked to ADPKD regardless of the presence or absence of PLD, while *PRKCSH* and *SEC63* variants are always associated with ADPLD without kidney cysts [24,25]. However, this separation is less obvious when minor genes such as *ALG8*, *GANAB*, or *LRP5* cause PLD [12,37,41,42]. Heterozygous pathogenic variants in these minor genes can cause ADPLD and ADPKD [24,25]. The current reported individuals diagnosed with a heterozygous pathogenic variant in *ALG9* developed polycystic kidneys and occasionally a polycystic liver as an extrarenal manifestation [12]. However, the diagnosis of a heterozygous pathogenic variant in *ALG9* in an ADPLD individual without kidney cysts indicates that pathogenic variants in this gene are not restricted to ADPKD development. Therefore, pathogenic variants in *ALG9* should be considered during the genetic screening of both ADPKD and ADPLD individuals.

Protein-truncating variants are generally associated with a more severe phenotype relative to missense variants. The currently described individuals with *ALG9*-caused conditions mainly had heterozygous or homozygous pathogenic missense variants (cysts and CDG patients) [10,12]. In contrast, *ALG8* variants that cause PLD are primarily heterozygous protein-truncating variants [32,34,59]. However, the phenotypic severity or inclination to only ADPKD or ADPLD development does not differ between these groups. This implies that even though both enzymes are essential for N-linked protein glycosylation, small conformational changes due to missense variants in *ALG9* are more damaging to a cell than in *ALG8*. The lack of protein-truncating variants in *ALG9* might even suggest that this type of variants in *ALG9* are often lethal.

Loss of heterozygosity (LOH) due to a second pathogenic variant, specifically in the liver or kidney, is often hypothesized to be involved in the molecular mechanism behind cyst development [60,61,62]. The original idea entailed the complete loss of expression of the protein of interest [60]. However, similar to our *ALG9* patient, this complete loss could not be proven in the cystic liver specimens of all patients [63]. However, in many of these cysts, large regions of copy-number-neutral (CNN) LOHs were found [64,65]. This implies that the second somatic variant can also occur at another genetic locus and that cyst development is initiated when these digenic pathogenic variants interrupt the interaction between its proteins. This disruption could explain the presence of ALG9 in the epithelium that lined the cyst of both ADPLD patients. The absence of ALG9 in the ADPKD patient’s cyst wall lining suggests that the progression of liver cyst growth in ADPLD patients is different than in ADPKD patients, and that this progression in ADPLD is due to a molecular mechanism that requires high levels of ALG9.

The discovery of the initial ADPLD genes was propelled by the availability of large families with a clearly defined clinical phenotype and performed through classical positional cloning. The discovery of the first two genes linked to ADPLD, *PRKCSH,* and *SEC63* was facilitated by the availability of large multiple-generation families [66,67,68,69,70]. From each ADPLD family, at least 5–15 affected individuals spread over different generations were accessible per family, and it was possible to assign the correct phenotype through (bedside) ultrasound [66,67,68,69,70]. The linkage strategy that was employed required multiple affected family members that could be sequenced to identify shared genetic variation.

However, the number of clinical observations suggests that the linkage strategy is less feasible nowadays. Most patients living with ADPLD who are diagnosed at this time and age give a negative family history and do not come from large families with readily available phenotypes and biomaterials. Indeed, many individuals seen in outpatient clinics are singletons without the availability of an extensive family or who come from families in whom the presence of liver and kidney cysts is simply unknown. This could be because these family members are genuinely unaffected, or the disease has not been penetrant at the time of ascertainment. In addition, assigning the correct phenotype (ADPLD or ADPKD) may be difficult in some cases. Apart from the presence of polycystic livers, many of these patients possess a number of (bilateral) renal cysts that technically meet the present Ravine criteria for ADPKD. However, their creatinine clearance is normal, and a family history for renal failure is absent. Indeed, there are families with a clearly affected index patient (either ADPLD or ADPKD) whose family members may be asymptomatic carriers of only a few liver or kidney cysts outside formal ADPKD or ADPLD criteria or, alternatively, families where the phenotypical spectra of affected members in that family may range from clearcut ADPKD to bona fide ADPLD.

The advent of next-generation sequencing has transformed Mendelian disease gene identification and probably surpassed traditional genetic linkage analysis. This has improved the laboratory workflow and prioritization of findings. However, correct adjudication of a gene to liver or kidney cyst development and of whether a specific variant is pathogenic and related to cystogenesis has become challenging. Due to the difficulty of performing co-segregation analyses due to small families, the identification of novel PLD genes has taken a new turn. There are a number of tools available, such as in silico and in vitro techniques for pathogenicity prediction, structural conformation determination, investigation of functional changes resulting from complete protein absence, and comparison of the prevalence of gene variants to the general population and PLD population. The improvement in these techniques and the advancement of high-throughput screening techniques have spurred the identification of novel ADPLD- and ADPKD-causing genes [26,30,35,43].

We think there are three key strategies specific to ADPLD that may offer additional help here. It is possible to search for variants in a single gene in multiple unrelated patients with a similar phenotype. While there is clear genetic heterogeneity, the number of unlinked patients is large. Unlike ADPKD, in which almost all patients receive a genetic diagnosis indicating pathogenic variants in *PKD1* and *PKD2*, the set of nine ADPLD-associated genes that have been recognized up to this point only explains 30–45% of the cases within the ADPLD population [16,24,25]. By increasing the pool of unlinked patients, it will be possible to enrich the pool of patients affected by a similar gene.

Gene identification may be facilitated by using the principle of the two-hit disease model [15,16,23,24,60,64,65]. This model proposes a second (somatic) variant and the resulting loss of heterozygosity as the mechanism underlying cyst formation [15,16,23,24,60,64,65]. Indeed, loss of heterozygosity has been shown for *PRKCSH-* and *SEC63*-affected ADPLD individuals [60,64,65]. There, the loss of the wild-type alleles from the cyst epithelium results in the absence of the protein expression from the cyst epithelium. The presence of biomaterials that may support the double-hit hypothesis (such as liver cyst lining) may aid in identifying the genetic locus.

Prioritization of genes that encode proteins located in the ER can benefit gene candidate strategies. In contrast to ADPKD, where genes encode proteins that localize to the primary cilium, the gene products implicated in ADPLD (*PRKCSH*, *SEC63*, *SEC61A*, *SEC61B*, *GANAB*, *ALG8*, and *ALG9*) are all involved in the co-translational translocation and maturation of glycoproteins in the endoplasmic reticulum [15,16,24]. These processes are important for the quality control of glycoproteins [71]. In the majority of secretory and membrane proteins, protein folding quality control in the ER is related to N-linked glycosylation [71]. It is reasonable to suggest that future gene discoveries will identify further genes encoding ER-resident proteins to be implicated in ADPLD.

The lack of genetic diagnosis in the majority of ADPLD individuals suggests that the number of genes associated with PLD will increase in the coming years [16,24,25]. However, at the moment, the exact understanding of the molecular mechanism underlying polycystic liver disease is still incomplete. The complex interaction between the already identified cellular mechanisms highlights the complexity of polycystic liver disease and presents attractive opportunities for specific treatment therapies intended to restore normal cellular function and to slow the progression of the disease.

In conclusion, pathogenic variants in *ALG9* are also associated with ADPLD without kidney cysts. This expands the clinical phenotype of heterozygous pathogenic *ALG9* variants and broadens the genotype–phenotype spectrum between ADPLD and ADPKD.

## Data Availability

Additional data are available from the corresponding author upon request.
